# Genetic diversity of *Bartonella* infection in residential and field rodents in Hebei, China

**DOI:** 10.3389/fmicb.2022.1039665

**Published:** 2022-11-25

**Authors:** Rui Jian, Qing Ren, Jing Xue, Guang-Cheng Xie, Jiangli Wang, Guo-Qing Chen, Luanying Du, Wen-Ping Guo

**Affiliations:** ^1^Department of Pathogenic Biology, College of Basic Medicine, Chengde Medical University, Chengde, Hebei, China; ^2^Laboratory of Microbiology Detection, Chengde Center for Disease Control and Prevention, Chengde, China; ^3^Yancheng Center for Disease Control and Prevention, Yancheng, China

**Keywords:** *Bartonella*, epidemiology, genetic diversity, human-pathogenic, rodents

## Abstract

Rodents are the primary natural reservoirs of *Bartonella* spp., and some of which are zoonotic causative agents. Hence, surveillance of *Bartonella* sp. infection in rodents is very important for the prevention of human bartonellosis caused by them. In this study, rodents were captured, and their spleen samples were collected for *Bartonella* sp. DNA detection and identification by amplifying the 16S rRNA, *gltA*, and *ftsz* genes using semi-nested polymerase chain reaction (PCR). The results indicated that *Bartonella* sp. DNA was detected in seven *Rattus norvegicus* individuals with a detection rate of 6.7% in Chengde City and bacterial DNA in 31 *Apodemus agrarius* individuals with a detection rate of 28.4% in Handan City. The DNA detection rate across the genders and ages of rodents was not found to be statistically significant. Furthermore, sequence analysis of the above-mentioned three genes demonstrated that at least eight *Bartonella* species were circulating in Hebei Province, of which three, including *Bartonella rattimassiliensis*, *Bartonella grahamii*, and *Bartonella tribocorum*, are human pathogens, thus suggesting the existence of a major public health risk. Overall, these results revealed the detection rate and genetic diversity of *Bartonella* species infection in rodents in Hebei Province, which could be potentially helpful for the prevention of bartonellosis caused by rodent-associated *Bartonella* species. This study highlights the urgent need for the surveillance of *Bartonella* infections in rodents and ectoparasites that affect both rodents and humans and can cause fever of unknown origin or endocarditis.

## Introduction

*Bartonella* spp. belong to the genus *Bartonella* within the family Bartonellaceae and are Gram-negative facultative intracellular alphaproteobacteria (Okaro et al., 2017). Before the reclassification of *Bartonella* in 1993, only one species, *B. bacilliformis*, was recorded. After that, the number of *Bartonella* spp. has continued to increase rapidly over the past 30 years, with currently more than 50 validated species and more than 10 candidate species (Okaro et al., 2017; Krügel et al., 2022). In addition, some complete genome sequences representing potential novel species are likely to further expand the number of species in the genus *Bartonella* (Lin et al., 2008; Sato et al., 2012). *Bartonella* spp. can infect a wide range of different domestic and wild animals, including cats, dogs, rodents, cattle, sheep, and bats (Okaro et al., 2017). Moreover, an increasing variety of animals have been confirmed as hosts of *Bartonella* spp., such as the beluga whale (Maggi et al., 2008), kangaroo (Fournier et al., 2007), camel (Rasis et al., 2014), and wild carnivores (Marciano et al., 2016; López-Pérez et al., 2017). *Bartonella* spp. are zoonotic bacteria and can be transmitted from animals to humans indirectly by blood-sucking arthropods (Deng et al., 2012), as well as through direct contact through the scratch of an infected cat or with the infected feces of a vector (Krügel et al., 2022). *Bartonella henselae* and *Bartonella quintana*, which mainly cause cat scratch disease (CSD) and trench fever in humans, respectively, have attracted more attention due to more patients being attributed as having the infections (Okaro et al., 2017). More seriously, at least another 18 species are considered to be human pathogens or have been identified in humans with the clinical symptom of fever (Maggi et al., 2009; Chomel and Kasten, 2010; Vayssier-Taussat et al., 2016; Okaro et al., 2017; Krügel et al., 2022).

Rodents are the natural reservoir of many human pathogens, including viruses, bacteria, and protozoans (Meerburg et al., 2009). There is no doubt that rodents play an important role in the maintenance and transmission of *Bartonella* spp., and at least 40 *Bartonella* species have been identified in a great diversity of rodents (Okaro et al., 2017; do Amaral et al., 2022; Krügel et al., 2022). Among them, eight have been proven to be pathogenic to humans and are hosted by rodents. These include *Bartonella doshiae* (Vayssier-Taussat et al., 2016), *Bartonella elizabethae* (Daly et al., 1993), *Bartonella grahamii* (Kerkhoff et al., 1999), *Bartonella rattimassiliensis* (Kosoy et al., 2010), *Bartonella rochalimae* (Eremeeva et al., 2007), *Bartonella tribocorum* (Kosoy et al., 2010), *Bartonella vinsonii* (Roux et al., 2000), and *Bartonella washoensis* (Kosoy et al., 2003). In addition, *B. henselae*, mainly hosted by cats, has also been detected in several rodent species, although their role in the maintenance and transmission of *B. henselae* remains unclear (Helan et al., 2018; Divari et al., 2020; Böge et al., 2021). In China, at least 22 species, including eight human pathogens, have been reported in rodents, with infection rates ranging from 6.4% to 57.7% to date (Saisongkorh et al., 2009; Li et al., 2015; Xia et al., 2015; Qin et al., 2019; Rao et al., 2021; Krügel et al., 2022; Yao et al., 2022). Therefore, we speculate that the Chinese population could be at risk of being affected by rodent-associated *Bartonella* spp., although human infections have not yet been identified.

Rodent-associated *Bartonella* spp. have been found to be widely distributed around the world, as well as in China, and a number of previous studies have mainly focused on the field environment (Okaro et al., 2017; Krügel et al., 2022). Hebei Province has a complex and varied terrain consisting of plateaus, mountains, and plains, which are beneficial to rodent colonization and survival. Moreover, closer contact between humans and rodents in residential areas, as well as diverse human activities that continue to invade the wild habitat of rodents, can significantly increase the transmission risk of *Bartonella* spp. from rodents to humans. Bartonellosis, which is caused by the rodent-associated *Bartonella* spp., is a natural focal disease, and thus investigations of the epidemiology and genetic diversity of *Bartonella* spp. infection in rodents can be of great significance for the prevention of human bartonellosis. Therefore, in this study, rodent samples were collected from Hebei Province to analyze the *Bartonella* sp. infection in the rodent populations, especially those pathogenic to humans. The results of this study will not only benefit the prevention and control of human bartonellosis in the local population but will also be helpful for the accurate diagnosis of diseases with fever of unknown origin (FUO).

## Materials and methods

### Rodent sample collection and deoxyribonucleic acid extraction

**FIGURE 1:**
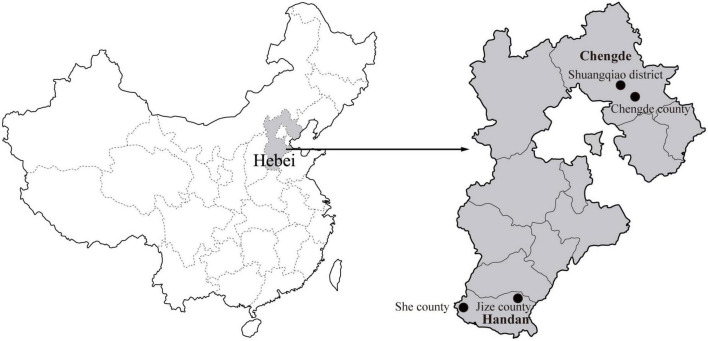
Geographical distribution of the rodent sampling sites (black dots) in Hebei Province, China. The map of China was obtained freely from http://english.freemap.jp/item/asia/china.html. The map of Hebei Province was generated using ArcGIS.

During 2020, rodents were captured using live capture traps baited with peanuts in residential areas of Chengde City and field areas of Handan City in Hebei Province, and two sampling sites were established in each city (Figure 1). The species, age, and sex of all the captured rodents were identified and recorded (Chen, 1987; Wang, 2003). All the captured rodents were euthanized, and the spleen specimens were aseptically collected.

Total DNA was extracted from the spleen samples using a DNA extraction kit (OMEGA, Doraville, CA, USA) in a fume hood in a separate room. The extracted DNA was eluted with 50 μl of double-distilled water and stored at−20°C before analysis. In addition, the identification of the rodent species was further confirmed by sequence analysis of the mt-*cyt* b gene obtained by PCR using the extracted DNA as the template (Guo et al., 2013).

### Detection and molecular characterization of *Bartonella* spp.

The presence of *Bartonella* spp. in rodents was detected by amplifying part of the 16S rRNA gene using a semi-nested polymerase chain reaction (PCR) and subsequently confirmed by further sequencing. The primer pairs Bar-SF1/Bar-SR and Bar-SF2/Bar-SR designed in this study were used for the first and second rounds of PCR to amplify an 813-bp 16S rRNA gene fragment.

In addition, the nearly complete 16S rRNA, the partial citrate synthase gene (*gltA*), and the cell division protein gene (*ftsZ*) were obtained from the *Bartonella* sp. DNA-detected samples to better determine and characterize the *Bartonella* species. Briefly, the primer pairs Bar-SF/Bar-SR1 and Bar-SF/Bar-SR2 were used to obtain the rest of the 16S rRNA with a length of 712 bp. The partial *gltA* gene (1,036 bp) was amplified with the primer pairs Bar-gltA-F/Bar-gltA-R1 and Bar-gltA-F/Bar-gltA-R2 for the primary and secondary rounds of the semi-nested PCR, respectively. The partial *ftsz* gene (860 bp) was amplified with the primer pairs Bar-ftsz-F1/Bar-ftsz-R and Bar-ftsz-F2/Bar-ftsz-R for the primary and secondary rounds of the semi-nested PCR, respectively. Alternatively, Bar-gltA-FM and Bar-ftsz-RM, instead of Bar-gltA-F and Bar-ftsz-R, were employed to amplify the shorter *gltA* (476 bp) and *ftsz* (575 bp) genes, respectively. All the primer sequences used in the present study are shown in Table 1.

**TABLE 1:** Primers used in this study.

Gene	Primers	Sequences (5′ → 3′)	Tm
16S rRNA	Bar-SF1	GGAAGAGGTGAGTGGAATTC	52°C
	Bar-SF2	GGAATTCCSAGTGTAGAGGT	
	Bar-SR	TCGCTGACCCTACCGTGGT	
	Bar-SF	CYGGCTCAGAACGAACGCTG	52°C
	Bar-SR1	CGTGGACTACCAGGGTATCT	
	Bar-SR2	GGTATCTAATCCTGTTTGCTC	
*gltA*	Bar-gltA-FM	GCHGATCAYGARCAAAATGC	54°C
	Bar-gltA-F	TTACYTAYGAYCCYGGBTTTA	
	Bar-gltA-R1	CYTCRATCATTTCTTTCCAYTG	
	Bar-gltA-R2	GCAAAVAGAACMGTRAACAT	
*Ftsz*	Bar-ftsz-F1	ATGACGATTAATCTGCATCG	50°C
	Bar-ftsz-F2	ATTAATCTGCATCGGCCAGA	
	Bar-ftsz-R	TCTTCRCGRATACGATTRGC	
	Bar-ftsz-RM	TAAAGHACTTGRTCAGCCAT	

The PCR reaction for the first round of the nested PCR was performed in a 20 μl reaction volume, containing 10 μl *Premix Taq* (Takara, Dalian, China), 1.5 μl extracted DNA, 0.8 μl of each primer (10 pmol), and 6.9 μl water. For the second round, the PCR mixture contained 25 μl *Premix Taq* (Takara, Dalian, China), 3 μl of the first-round PCR products, 2 μl of each primer (10 pmol), and 18 μl water for a final volume of 50 μl. The same thermal cycling condition was used for both rounds, including a denaturation at 94°C for 5 min, 30 cycles of denaturation at 94°C for 40 s, annealing at 56°C for 40 s, and elongation at 72°C for 1 min, followed by a final extension at 72°C for 7 min. In addition, to prevent contamination, the PCR mixture preparation, template addition, and agarose gel electrophoresis were performed in a fume hood in three separate rooms, and filter tips were also used in each assay. Furthermore, ddH_2_O was used as a negative control.

The PCR product was electrophoresed on a 1.0% agarose gel, and the amplicon of the expected size was purified using the Takara MiniBEST Agarose Gel DNA Extraction Kit Version 4.0 (Takara, Dalian, China). The purified PCR product was subjected to bidirectional sequencing with the PCR primers. Alternatively, the purified PCR product was ligated into the pGEM-T vector for sequencing when it produced complex sequence chromatograms in direct sequencing. The PCR products were sequenced using the ABI-PRISM Dye Termination Sequencing Kit and the ABI 3730 genetic analyzer. All the newly generated sequences in this study were submitted to GenBank under the accession numbers OP363479-OP363516 for the *rrs* gene, OP382327-OP382454 for the *gltA* gene, and OP382391-OP382426 for the *ftsZ* gene.

### Sequence analysis

The nearly complete 16S rRNA gene was assembled by partially overlapping oligonucleotides using BioEdit version 7.1.11 (Hall, 1999). The nucleotide identities of the 16S rRNA, *gltA*, and *ftsZ* genes were calculated using the MegAlign program within the DNASTAR Lasergene software (Burland, 2000). Phylogenetic trees based on the 16S rRNA, *gltA*, and *ftsZ* gene sequences were reconstructed using the maximum-likelihood (ML) method within PhyML version 3.2, and the reliability of branches in the tree was evaluated by calculating the bootstrap values with 1,000 replicates (Guindon et al., 2010). The general time-reversible (GTR) substitution model with a gamma (Γ) model of rate heterogeneity and a proportion of invariable sites (GTR + Γ + I) determined by MEGA 7.0 was found to be the best fit model for the phylogenetic analysis (Kumar et al., 2016). The ML tree was rooted at its midpoint using MEGA 7.0.

### Statistical analyses

The *P*-value with the chi-square test was calculated using SPSS 21.0 software for the statistical analyses to determine the association between the detection rate and the gender and age of the rodents. A *P*-value of less than 0.05 was considered to be statistically significant.

### Ethics statement

This study was approved by the ethics committee of the Chengde Medical University (No. 202004). All the rodent experiments were performed in strict accordance with the Guidance for Experimental Animal Welfare and Ethical Treatment by the Ministry of Science and Technology of China. In addition, the rodents were anesthetized with ether and dissected to collect spleen samples for the detection of *Bartonella* sp. DNA, and analgesics were administered to minimize the suffering of the captured rodents.

## Results

### Sample collection and detection of *Bartonella* sp. deoxyribonucleic acid in rodents

A total of 223 rodents were captured from four sampling sites in Chengde and Handan cities in Hebei Province, China (Table 2). The collected rodents were identified into five distinct species comprising *Rattus norvegicus*, *R. tanezumi*, *Mus musculus*, *Apodemus agrarius*, and *Cricetulus triton*. In Chengde City, the rodents were sampled in residential areas, and *R*. *norvegicus* was found to be the most abundant species, while *A*. *agrarius* was the predominant species in Handan, where it was captured in the field.

**TABLE 2:** *Bartonella* sp. DNA detection in rodents collected in Hebei, China.

Parameters	Location				Total
	Chengde City		Handan City		
	Shuangqiao District	Chengde County	Jize County	She County	
**Species**					
*R. norvegicus*	4/48	3/43	0/2	0/1	7/94
*R. tanezumi*	0/2	0/3	–	–	0/5
*M. musculus*	0/1	0/5	0/3	0/1	0/10
*A.agrarius*	–	0/2	12/49	19/51	31/102
*C. triton*	–	–	–	0/2	0/2
**Gender**					
Female	1/11	2/38	4/20	8/21	15/90 (95% CI: 4.3-29.1)
Male	3/40	1/15	8/34	11/34	23/133 (95% CI: 6.0-27.4)
**Age**					
Juvenile	2/24	2/41	3/17	5/15	12/97 (95% CI: 5.5-26.8)
Adult	2/37	1/12	9/37	14/40	26/126 (95% CI: 6.5-30.1)

Based on the amplification of the 16S rRNA gene, PCR products of the expected sizes were detected in 38 spleen samples collected from the 223 rodents. After sequencing of the PCR products, all the obtained 16S rRNA gene sequences were blasted against the GenBank nucleotide database, and the results showed that all of them shared more than 99.4% nucleotide identity with the most closely related 16S rRNA gene sequence of a *Bartonella* species. Hence, the DNA detection rate was found to be 17.0%. Specifically, seven of the *Bartonella* sp. DNA-detected samples were identified as being from *R*. *norvegicus* in Chengde with a detection rate of 6.7% and 31 from *A*. *agrarius* in Handan with a detection rate of 28.4%. Furthermore, the infection rates in female and male rodents were observed to be 16.7% (95% CI: 4.3–29.1%) and 17.3% (95% CI: 6.0–27.4%), respectively, which were not statistically significant (χ^2^ = 0.808, *P* = 0.567). Similarly, no significant difference (χ^2^ = 0.887, *P* = 0.538) was noted in the infection rates between juvenile (12.4%, 95% CI: 5.5–26.8%) and adult rodents (20.6%, 95% CI: 6.5–30.1%).

### Molecular characterization of the *Bartonella* species

To better identify and characterize the *Bartonella* species in the current study, 37 nearly complete 16S rRNA gene sequences (with a length of approximately 1,400 bp), 28 partial *gltA* gene sequences (16 with a length of approximately 1,000 bp and 12 others with a length of approximately 440 bp), and 36 partial *ftsZ* gene sequences (31 with a length of approximately 830 bp and 5 others with a length of approximately 550 bp) were obtained from all the samples with *Bartonella* sp.-DNA detection. The sequencing and BLAST analyses based on three gene sequences indicated that all the *Bartonella* strains identified in this study were classified into at least eight different *Bartonella* species, namely, *B. japonica*, *Bartonella* sp. 1-1C (a *B. rochalimae*-like species), *B. rattimassiliensis*, *B. grahamii*, *B. taylorii*, *B. tribocorum*, *B. mastomydis*, and *B. kosoyi*. In addition, a 770-bp 16S rRNA gene sequence of Shexian-Aa46 from *A*. *agrarius* in Handan shared the highest nucleotide identity with *B. vinsonii* (99.5%), while its *ftsZ* gene exhibited the highest nucleotide identity with that of *Bartonella* sp. 1-1C (99.6%), which might have been caused by coinfection with two different *Bartonella* species. Due to a failure to obtain the *gltA* and *ftsZ* gene sequences resembling those of *B. vinsonii* from Shexian-Aa46, *B. vinsonii* or *B. vinsonii*-like bacteria could have been circulating in *A*. *agrarius* in local areas.

Specifically, five distinct *Bartonella* species, including *B. japonica* (n = 1), *Bartonella* sp. 1-1C (*n* = 2), *B. rattimassiliensis* (*n* = 2), *B. grahamii* (*n* = 1) and *B. taylorii* (*n* = 1), were detected in *R*. *norvegicus* in Chengde City. In Handan City, *Bartonella* sp. 1-1C, *B. tribocorum*, *B. mastomydis*, and *B. kosoyi* were identified from 6, 10, 1, and 12 *A*. *agrarius*, respectively. For Jize-Aa8 belonging to *B. mastomydis* in *A*. *agrarius*, only an approximate 1,000-bp *gltA* gene sequence was obtained, which shared the highest nucleotide identity of 99.4% with that of the *B. mastomydis* strain 008 (KY555066), while its 1,400-bp 16S rRNA belonged to *B. tribocorum*, which also may have been potentially caused by co-infection with *B. mastomydis* and *B. tribocorum*. Of the eight *Bartonella* species, only one, *Bartonella* sp. 1-1C, was detected in both *R*. *norvegicus* in Chengde City and *A*. *agrarius* in Handan City.

The strain Chengde-Rn16 shared the highest nucleotide identities of 99.6% and 97.9% with the *B. japonica* strain Fuji 18-1 for the 16S rRNA and *gltA* genes, respectively. The strain Chengde-Rn85 shared the highest nucleotide identities of 100%, 99.6%, and 99.6% with the *B. grahamii* strain as4aup for the 16S rRNA, *gltA*, and *ftsz* genes, respectively. The strain Chengde-Rn40 showed the highest nucleotide identity of 100% with the *B. taylorii* strain IBS296 for the 16S rRNA, 100% with the *B. taylorii* strain Far East I for the *gltA*, and 98.9% with the *B. taylorii* strain M1 for the *ftsz* genes. The two *B. rattimassiliensis* strains, Shuangqiao-Rn48 and Chengde-Rn25, displayed the highest nucleotide identities of 99.9% and 99.9%, respectively, with the *B. rattimassiliensis* strain 15908 for the 16S rRNA and *ftsz* genes, and 99.1% and 98.9%, respectively, with the *B. rattimassiliensis* strain SD-10 for the *gltA* gene. The seven *Bartonella* sp. 1-1C strains identified here shared 99.8–100%, 98.9–100%, and 97.6–100% nucleotide identities with each other and the highest nucleotide identities of 99.9–100%, 99.3–100%, and 97.0–100% with the *Bartonella* sp. 1-1C for the 16S rRNA, *gltA*, and *ftsz* genes, respectively. The 10 *B. tribocorum* strains identified here shared 99.9–100%, 99.3–100%, and 99.5–100% nucleotide identities with each other and the highest nucleotide identities of 99.9%, 99.6–99.0%, and 99.6–100% with the known *B. tribocorum* strains for the 16S rRNA, *gltA*, and *ftsz* genes, respectively. The 13 *B. kosoyi* strains identified here shared 99.6–100%, 99.5–99.8%, and 99.8–100% nucleotide identities with each other and the highest nucleotide identities of 99.7–100%, 99.6–99.8%, and 99.8–100% with the *B. kosoyi* strain Tel Aviv for the 16S rRNA, *gltA*, and *ftsz* genes, respectively.

**FIGURE 2:**
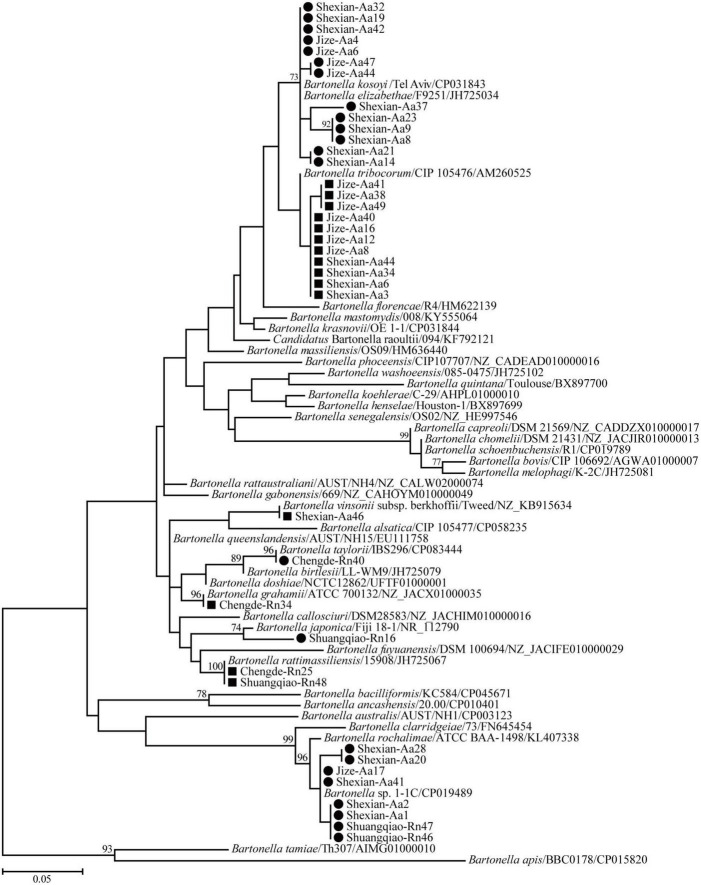
Maximum-likelihood (ML) tree reconstructed based on the 16S rRNA gene sequences of *Bartonella* species. Bootstrap values were calculated with 100 replicates and only > 70% are shown. Sequences of human-pathogenic *Bartonella* species determined herein are marked as black squares and others as black circles.

**FIGURE 3:**
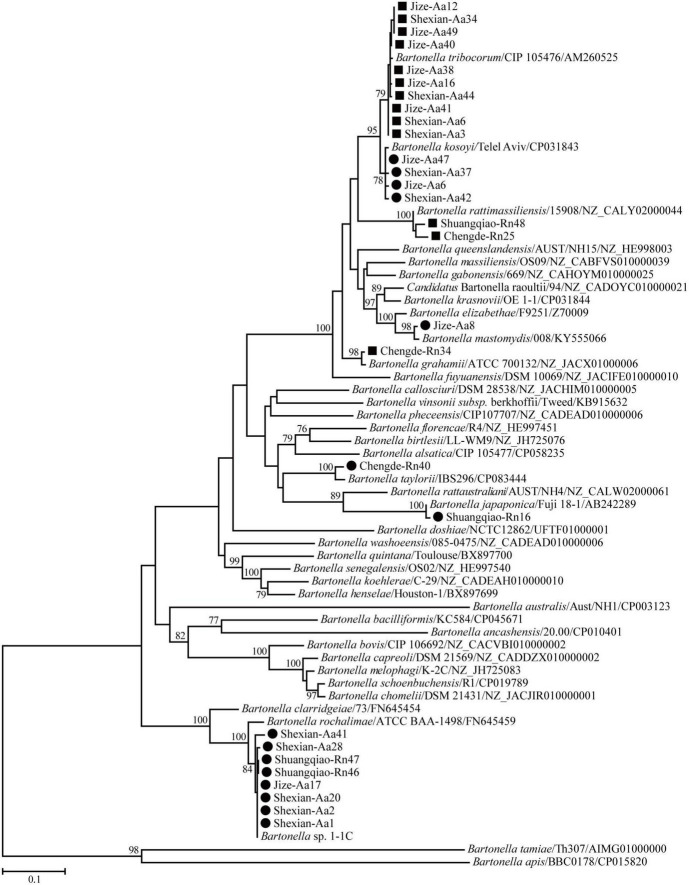
Maximum-likelihood (ML) tree reconstructed based on the *gltA* gene sequences of *Bartonella* species. The legend follows that of Figure 2.

**FIGURE 4:**
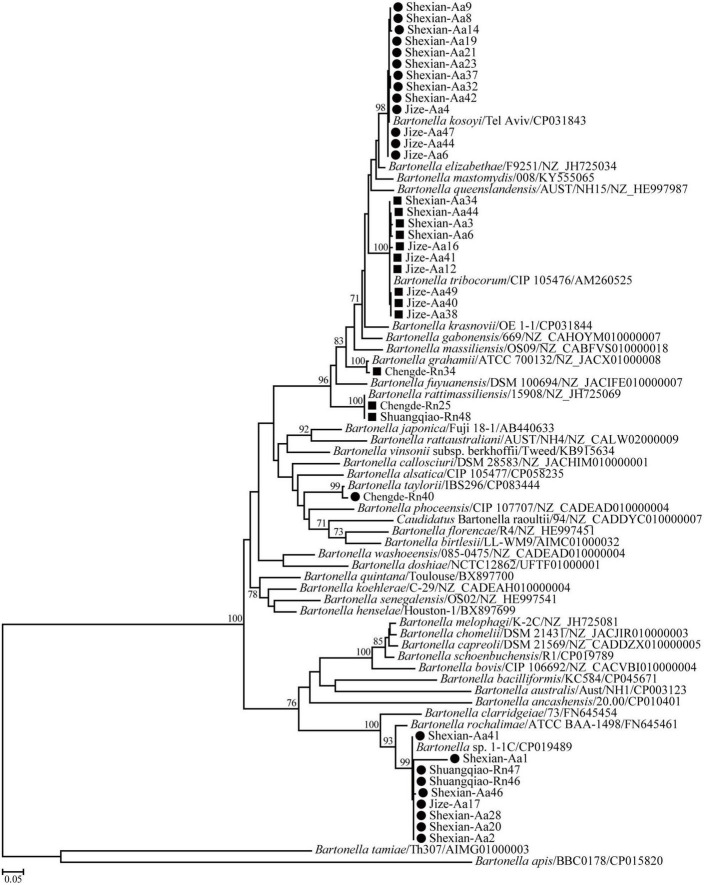
Maximum-likelihood (ML) tree reconstructed based on the *ftsz* gene sequences of *Bartonella* species. The legend follows that of Figure 2.

Phylogenetic trees based on the 16S rRNA, *gltA*, and *ftsz* gene sequences were reconstructed, and these three trees presented similar topologies. Consistent with the genetic analysis, all sequences in the three trees were divided into eight different groups that corresponded to *B. japonica*, *Bartonella* sp. 1-1C, *B. rattimassiliensis*, *B. grahamii*, *B. taylorii*, *B. tribocorum*, *B. mastomydis*, and *B. kosoyi* (Figures 2-4). Moreover, the 16S rRNA gene sequence of Shexian-Aa46 clustered with that of *B. vinsonii* in the 16S rRNA tree (Figure 2). However, in the phylogenetic tree of *ftsz*, all sequences obtained in this study were classified into six different groups due to the absence of the *ftsz* gene sequence in the *B. japonica* strain Chengde-Rn16 and the *B. mastomydis* strain Handan-Aa8.

## Discussion

Over the past 30 years, an increasing number of rodent-borne *Bartonella* species and associated patients with fever or endocarditis have been reported, suggesting more common human exposures to these agents than previously suspected (Daly et al., 1993; Kerkhoff et al., 1999; Roux et al., 2000; Kosoy et al., 2003, 2010; Eremeeva et al., 2007; Vayssier-Taussat et al., 2016; Okaro et al., 2017; Krügel et al., 2022). In addition, some species that were not previously thought to infect humans have now been confirmed to be human pathogens (Eremeeva et al., 2007; Vayssier-Taussat et al., 2016; Krügel et al., 2022). Hence, human-pathogenic *Bartonella* spp. are being considered emerging zoonotic causative agents, and more attention should be paid to their infection in reservoirs, vectors, and humans for the better prevention of bartonellosis.

Rodents are considered to be the primary natural hosts of *Bartonella* spp., and most known species have been detected in a wide variety of rodents. In China, more than half of the rodent-associated *Bartonella* species have been identified from rodents in residential and field areas (Saisongkorh et al., 2009; Xia et al., 2015; Rao et al., 2021; Krügel et al., 2022). *R. norvegicus* and *A. agrarius* are the predominant species found in residential areas and the wild, respectively, and they can also act as the hosts of *Bartonella* spp. In previous studies, at least seven validated *Bartonella* species, namely, *B. elizabethae*, *B. grahamii*, *B. rattimassiliensis*, *B. rochalimae*, *B. tribocorum*, *B. mastomydis*, and *B. queenslandensis*, were identified in *R. norvegicus* (Qin et al., 2019; An et al., 2020; Liu et al., 2022; Yao et al., 2022; Yu et al., 2022c), and six species, namely, *B. fuyuanensis*, *B. grahamii*, *B. phoceensis*, *B. japonica*, *B. taylorii*, and *B. coopersplainsensis*, were identified in *A. agrarius* (Li et al., 2015; Qin et al., 2019; Lu et al., 2022; Yu et al., 2022b). In the present study, eight official or candidate *Bartonella* species, in addition to *B. vinsonii*-like species, were identified in rodents. The findings indicated that genetically diverse *Bartonella* species are circulating in rodent populations in Hebei Province, China. In addition, this was the first identification of *B. kosoyi* in China.

Consistent with the previous study, no significant difference in the detection rate was associated with either the gender or the age of the rodents (Yao et al., 2022). In this study, it was found that the overall detection rate of *Bartonella* sp. infection in rodents was 17.0%, lower than that in rodents from the Qinghai-Tibetan Plateau (30.1%, Yu et al., 2022a), the Shangdang Basin (37.4%, Yu et al., 2022b), the Qaidam Basin (38.6%, Rao et al., 2021), Heixiazi Island (57.7%, Li et al., 2015), Shaanxi (26.1%, An et al., 2020), Zhongtiao Mountain (49.5%, Yu et al., 2022c), Zhejiang (31.4%, Liu et al., 2010), and South China (43.5%, Ying et al., 2002), while higher than that in rodents from eastern China (8.4%, Qin et al., 2019) and Guangzhou (6.4%, Yao et al., 2022). In addition, similar detection rates to those found in the present study were observed in rodents from Guizhou (16.1%; Lu et al., 2022) and southeastern China (14.9%; Liu et al., 2022). The discrepancy might have been due to the rodent species, habitats, and arthropod vector populations (Meheretu et al., 2013). In addition, compared to rodents collected in residential areas, a higher detection rate was observed in rodents collected in the wild (Yu et al., 2022b). Similarly, in this study, a *Bartonella* sp. DNA detection rate of 28.4% was observed in *R. norvegicus* in residential areas, while a 6.7% rate was found in *A. agrarius* in the field. However, *R. norvegicus* and *A. agrarius* were captured from different areas; thus, it was not clear whether the rodent species or the habitat was the factor that affected the detection rate, which is a limitation of this study.

In China, all eight rodent-related *Bartonella* species pathogenic to humans have been identified, mostly in the past 10 years (Li et al., 2015; Su et al., 2020; Yao et al., 2022). In this study, three human-pathogenic *Bartonella* species were detected, including *B. rattimassiliensis* and *B. grahamii* in Chengde and *B. tribocorum* in Handan. More importantly, the presence of *B. tribocorum* should be of concern due to the high detection rate of *A. agrarius*. In addition, attention should be paid to *B. rattimassiliensis* and *B. grahamii* due to the close association between their host, *R. norvegicus*, and humans, though they displayed a relatively low detection rate in rodents. *Bartonella* spp. are mainly transmitted from rodents to humans by blood-sucking arthropod vectors (Deng et al., 2012). In China, diverse *Bartonella* spp. have been identified in ectoparasites such as ticks, lice, and fleas (Li et al., 2007, 2013; Hao et al., 2020). Unfortunately, we failed to collect ectoparasites from the sampled rodents, which is another limitation of our study. Therefore, ectoparasites should be collected and analyzed in future studies. Furthermore, the presence of *Bartonella* spp. should be monitored and investigated in humans with fever of unknown origin or endocarditis in the local population.

As the natural reservoir hosts of many different types of microorganisms, rodents are considered to have a long-term co-divergence or co-speciation with the pathogens they carry, such as hantaviruses (Guo et al., 2013) and arenaviruses (Gonzalez et al., 2007). In terms of *Bartonella* species, Kosoy et al. also considered that they present potential co-evolution or co-speciation with their rodent hosts according to the host specificity (Kosoy et al., 2000). In addition, some previous studies have also demonstrated the host specificity of *Bartonella* sp. infection in rodents (Telfer et al., 2007; Abreu-Yanes et al., 2018; Qin et al., 2019; Divari et al., 2021). However, an increasing amount of evidence has suggested a lack of host specificity because the same *Bartonella* species can be identified from a diversity of rodent hosts, even from those belonging to different families (Billeter et al., 2014; Krügel et al., 2022). In the current study, *B. japonica*, *B. rattimassiliensis*, *B. grahamii*, and *B. taylorii* only in *R*. *norvegicus* and *B. tribocorum*, *B. mastomydis*, and *B. kosoyi* only in *A*. *agrarius* appear to support the view that *Bartonella* infection in rodents can exhibit host specificity. However, the *R*. *norvegicus* and *A*. *agrarius* in this study were sampled from different cities. In addition, the *Bartonella* species identified in this study were found to be carried by various rodents, as shown in previous studies. Hence, the apparent host specificity observed in this study might have been caused by the different habitats of the rodents.

## Conclusion

At least eight *Bartonella* species, including three human causative agents, were identified in two rodent species in Hebei Province, China. In addition, *Bartonella* sp. DNA detection rates of 28.4% and 6.7% were observed in *A*. *agrarius* in Handan City and in *R*. *norvegicus* in Chengde City, respectively. Our results also indicated a lack of host specificity for *Bartonella* sp. infection in rodents. Overall, our results indicate that more attention should be paid to the surveillance of rodent-associated *Bartonella* species.

## Data availability statement

The datasets presented in this study can be found in online repositories. The names of the repository/repositories and accession number(s) can be found in the article/Supplementary material.

## Ethics statement

This animal study was reviewed and approved by Ethics Committee of the Chengde Medical University.

## Author contributions

W-PG conceived and designed the experiments and contributed to writing, reviewing, and editing the manuscript. RJ, QR, JX, G-CX, G-QC, and LD performed the experiments and analyzed the data. W-PG and JW helped to collect the samples. W-PG and RJ participated in writing the original draft. All authors contributed to the article and approved the submitted version.
